# Supplementary material for: Hot spot prediction in protein-protein interactions by an ensemble system
Source: BMC Syst Biol. 2018 Dec 31;12(Suppl 9):132. doi: 10.1186/s12918-018-0665-8 (PMC6311905; doi:10.1186/s12918-018-0665-8)
Supplement: Supplementary file 2 — All relevant MATLAB code. A simple Matlab implement of our predictor. (ZIP 19,742 kb) [file 12918_2018_665_MOESM2_ESM.zip › Additional file 2/Read ASA/ASA Property/BID/1MQ8B.txt]

 NetSurfP - Protein Surface Accessibility and Secondary Structure Predictions

  

|  |  |  |
| --- | --- | --- |
|  |  | NetSurfP - Protein Surface Accessibility and Secondary Structure Predictions  Technical University of Denmark |

  


---

```
# For publication of results, please cite:
# A generic method for assignment of reliability scores applied to solvent accessibility predictions.
# Bent Petersen, Thomas Nordahl Petersen, Pernille Andersen, Morten Nielsen and Claus Lundegaard
# BMC Structural Biology 2009, 9:51 doi:10.1186/1472-6807-9-51
#
# Column 1: Class assignment - B for buried or E for Exposed - Threshold: 25% exposure, but not based on RSA
# Column 2: Amino acid
# Column 3: Sequence name
# Column 4: Amino acid number
# Column 5: Relative Surface Accessibility - RSA
# Column 6: Absolute Surface Accessibility
# Column 7: Z-fit score for RSA prediction
# Column 8: Probability for Alpha-Helix
# Column 9: Probability for Beta-strand
# Column 10: Probability for Coil
E V  1MQ8B                  1    0.296  45.526  -0.038   0.005   0.015   0.979
B D  1MQ8B                  2    0.241  34.728   0.120   0.002   0.816   0.182
B L  1MQ8B                  3    0.055  10.034   1.025   0.001   0.959   0.040
B V  1MQ8B                  4    0.038   5.917   0.046   0.000   0.983   0.017
B F  1MQ8B                  5    0.032   6.442   0.878   0.000   0.983   0.017
B L  1MQ8B                  6    0.024   4.431   1.196   0.000   0.983   0.017
B F  1MQ8B                  7    0.032   6.382   0.783   0.001   0.959   0.040
B D  1MQ8B                  8    0.070  10.159  -0.107   0.004   0.616   0.381
B G  1MQ8B                  9    0.191  15.040  -0.284   0.019   0.141   0.840
B S  1MQ8B                 10    0.104  12.154  -0.795   0.018   0.047   0.935
E M  1MQ8B                 11    0.408  81.601  -0.873   0.018   0.019   0.964
B S  1MQ8B                 12    0.262  30.671  -1.433   0.018   0.019   0.964
B L  1MQ8B                 13    0.162  29.662  -0.569   0.018   0.019   0.964
E Q  1MQ8B                 14    0.434  77.548   0.255   0.018   0.019   0.964
E P  1MQ8B                 15    0.582  82.643  -0.224   0.600   0.003   0.397
E D  1MQ8B                 16    0.673  96.994   0.424   0.502   0.002   0.495
E E  1MQ8B                 17    0.302  52.707   1.036   0.600   0.003   0.397
B F  1MQ8B                 18    0.059  11.922   0.851   0.923   0.002   0.076
E Q  1MQ8B                 19    0.556  99.284   1.868   0.970   0.001   0.030
E K  1MQ8B                 20    0.300  61.648   2.272   0.970   0.001   0.030
B I  1MQ8B                 21    0.020   3.681   1.171   0.970   0.001   0.030
B L  1MQ8B                 22    0.157  28.673   1.412   0.970   0.001   0.030
E D  1MQ8B                 23    0.512  73.851   1.855   0.970   0.001   0.030
B F  1MQ8B                 24    0.046   9.252   0.926   0.988   0.000   0.012
B M  1MQ8B                 25    0.014   2.881   1.369   0.988   0.000   0.012
E K  1MQ8B                 26    0.293  60.311   1.954   0.988   0.000   0.012
E D  1MQ8B                 27    0.402  57.914   2.185   0.988   0.000   0.012
B V  1MQ8B                 28    0.044   6.794   0.342   0.970   0.001   0.030
B M  1MQ8B                 29    0.039   7.764  -0.237   0.970   0.001   0.030
E K  1MQ8B                 30    0.545 112.086   1.830   0.923   0.002   0.076
E K  1MQ8B                 31    0.467  96.062   1.616   0.782   0.003   0.216
B C  1MQ8B                 32    0.069   9.674  -0.134   0.502   0.002   0.495
E S  1MQ8B                 33    0.362  42.380   0.303   0.109   0.005   0.886
E N  1MQ8B                 34    0.574  84.004   0.105   0.053   0.005   0.942
E T  1MQ8B                 35    0.674  93.442  -0.109   0.018   0.019   0.964
E S  1MQ8B                 36    0.355  41.641  -0.453   0.005   0.015   0.979
B Y  1MQ8B                 37    0.093  19.895  -0.227   0.005   0.262   0.733
B Q  1MQ8B                 38    0.108  19.235   0.469   0.002   0.816   0.182
B F  1MQ8B                 39    0.023   4.516   1.004   0.001   0.959   0.040
B A  1MQ8B                 40    0.021   2.358   0.837   0.001   0.959   0.040
B A  1MQ8B                 41    0.019   2.094   1.322   0.001   0.959   0.040
B V  1MQ8B                 42    0.025   3.858   0.862   0.001   0.959   0.040
B Q  1MQ8B                 43    0.103  18.449   0.501   0.001   0.959   0.040
B F  1MQ8B                 44    0.059  11.741   0.372   0.001   0.900   0.099
B S  1MQ8B                 45    0.150  17.568  -0.591   0.004   0.138   0.858
E T  1MQ8B                 46    0.476  66.077  -0.841   0.005   0.015   0.979
E S  1MQ8B                 47    0.479  56.092   0.567   0.005   0.336   0.660
B Y  1MQ8B                 48    0.097  20.622  -0.186   0.004   0.616   0.381
E K  1MQ8B                 49    0.284  58.398   0.633   0.018   0.846   0.136
B T  1MQ8B                 50    0.244  33.884   0.382   0.001   0.959   0.040
B E  1MQ8B                 51    0.253  44.269   0.254   0.001   0.900   0.099
B F  1MQ8B                 52    0.074  14.892   0.704   0.003   0.718   0.279
E D  1MQ8B                 53    0.361  52.049   0.050   0.004   0.420   0.576
B F  1MQ8B                 54    0.092  18.484  -1.162   0.004   0.085   0.910
E S  1MQ8B                 55    0.421  49.388  -0.458   0.005   0.015   0.979
E D  1MQ8B                 56    0.532  76.704  -0.059   0.018   0.019   0.964
E Y  1MQ8B                 57    0.327  69.816  -1.246   0.321   0.003   0.675
E V  1MQ8B                 58    0.529  81.353  -1.061   0.406   0.004   0.590
E K  1MQ8B                 59    0.478  98.283  -0.219   0.246   0.004   0.750
E R  1MQ8B                 60    0.245  56.151   0.798   0.923   0.002   0.076
E K  1MQ8B                 61    0.592 121.733   0.342   0.970   0.001   0.030
E D  1MQ8B                 62    0.416  60.018   1.580   0.970   0.001   0.030
B P  1MQ8B                 63    0.047   6.726   0.224   0.970   0.001   0.030
E D  1MQ8B                 64    0.266  38.359   0.965   0.970   0.001   0.030
E A  1MQ8B                 65    0.496  54.637   1.386   0.970   0.001   0.030
B L  1MQ8B                 66    0.187  34.295   0.233   0.923   0.002   0.076
B L  1MQ8B                 67    0.059  10.803  -0.386   0.858   0.002   0.139
E K  1MQ8B                 68    0.562 115.562   0.664   0.782   0.003   0.216
E H  1MQ8B                 69    0.567 103.210   1.040   0.600   0.003   0.397
B V  1MQ8B                 70    0.076  11.681  -0.114   0.430   0.016   0.555
E K  1MQ8B                 71    0.446  91.804   0.159   0.339   0.016   0.645
E H  1MQ8B                 72    0.376  68.322  -1.000   0.339   0.016   0.645
E M  1MQ8B                 73    0.401  80.280  -1.129   0.181   0.016   0.803
E L  1MQ8B                 74    0.528  96.732  -2.494   0.053   0.005   0.942
E L  1MQ8B                 75    0.369  67.637  -1.995   0.053   0.005   0.942
B L  1MQ8B                 76    0.173  31.621  -1.854   0.115   0.016   0.868
B T  1MQ8B                 77    0.059   8.239  -1.838   0.181   0.016   0.803
B N  1MQ8B                 78    0.145  21.213  -0.071   0.522   0.016   0.462
B T  1MQ8B                 79    0.039   5.409  -0.463   0.938   0.007   0.055
B F  1MQ8B                 80    0.098  19.709  -0.896   0.970   0.001   0.030
E G  1MQ8B                 81    0.261  20.501   0.633   0.970   0.001   0.030
B A  1MQ8B                 82    0.020   2.204   0.011   0.988   0.000   0.012
B I  1MQ8B                 83    0.030   5.457   0.317   0.988   0.000   0.012
E N  1MQ8B                 84    0.305  44.637   1.548   0.988   0.000   0.012
E Y  1MQ8B                 85    0.247  52.698   1.439   0.988   0.000   0.012
B V  1MQ8B                 86    0.020   3.043   0.636   0.970   0.001   0.030
B A  1MQ8B                 87    0.133  14.657   0.548   0.970   0.001   0.030
E T  1MQ8B                 88    0.532  73.844   1.313   0.970   0.001   0.030
E E  1MQ8B                 89    0.269  47.012   1.226   0.923   0.002   0.076
B V  1MQ8B                 90    0.045   6.870  -0.209   0.858   0.002   0.139
E F  1MQ8B                 91    0.283  56.778   0.404   0.782   0.003   0.216
E R  1MQ8B                 92    0.577 132.179   0.594   0.694   0.003   0.303
E E  1MQ8B                 93    0.487  85.114  -0.660   0.502   0.002   0.495
E E  1MQ8B                 94    0.605 105.659  -1.319   0.406   0.004   0.590
E L  1MQ8B                 95    0.539  98.618  -1.028   0.321   0.003   0.675
E G  1MQ8B                 96    0.569  44.780  -1.552   0.176   0.004   0.820
E A  1MQ8B                 97    0.456  50.273  -1.977   0.115   0.016   0.868
E R  1MQ8B                 98    0.328  75.020  -0.709   0.058   0.017   0.925
E P  1MQ8B                 99    0.653  92.661  -1.246   0.058   0.017   0.925
E D  1MQ8B                100    0.602  86.791  -1.133   0.018   0.019   0.964
B A  1MQ8B                101    0.180  19.847  -0.477   0.018   0.019   0.964
E T  1MQ8B                102    0.321  44.467   0.750   0.004   0.138   0.858
B K  1MQ8B                103    0.159  32.686   1.293   0.004   0.616   0.381
B V  1MQ8B                104    0.039   5.994   0.743   0.001   0.900   0.099
B L  1MQ8B                105    0.012   2.270   1.817   0.001   0.959   0.040
B I  1MQ8B                106    0.014   2.627   1.846   0.000   0.983   0.017
B I  1MQ8B                107    0.013   2.423   1.761   0.001   0.959   0.040
B I  1MQ8B                108    0.019   3.589   1.333   0.001   0.900   0.099
B T  1MQ8B                109    0.029   4.064   0.075   0.003   0.718   0.279
B D  1MQ8B                110    0.147  21.168   0.265   0.004   0.138   0.858
B G  1MQ8B                111    0.149  11.726  -0.860   0.005   0.015   0.979
E E  1MQ8B                112    0.454  79.244   0.629   0.005   0.015   0.979
B A  1MQ8B                113    0.238  26.195  -1.165   0.018   0.019   0.964
E T  1MQ8B                114    0.465  64.509  -1.429   0.053   0.005   0.942
E D  1MQ8B                115    0.530  76.315  -1.071   0.176   0.004   0.820
E S  1MQ8B                116    0.412  48.228  -0.438   0.502   0.002   0.495
B G  1MQ8B                117    0.137  10.782  -0.639   0.782   0.003   0.216
E N  1MQ8B                118    0.580  84.853   0.960   0.782   0.003   0.216
E I  1MQ8B                119    0.423  78.274   1.345   0.858   0.002   0.139
B D  1MQ8B                120    0.142  20.491  -0.425   0.858   0.002   0.139
E A  1MQ8B                121    0.463  51.078   0.797   0.782   0.003   0.216
E A  1MQ8B                122    0.673  74.176   0.450   0.600   0.003   0.397
E K  1MQ8B                123    0.480  98.654   1.029   0.053   0.005   0.942
E D  1MQ8B                124    0.695 100.106   0.724   0.005   0.015   0.979
B I  1MQ8B                125    0.038   7.104   0.499   0.005   0.262   0.733
E I  1MQ8B                126    0.312  57.775   1.853   0.001   0.900   0.099
B R  1MQ8B                127    0.037   8.450   0.341   0.001   0.959   0.040
B Y  1MQ8B                128    0.046   9.873   1.074   0.000   0.983   0.017
B I  1MQ8B                129    0.017   3.219   1.025   0.000   0.983   0.017
B I  1MQ8B                130    0.023   4.163   1.148   0.001   0.959   0.040
B G  1MQ8B                131    0.020   1.574  -0.157   0.001   0.900   0.099
B I  1MQ8B                132    0.039   7.252   0.285   0.004   0.616   0.381
B G  1MQ8B                133    0.077   6.028  -1.540   0.004   0.197   0.799
E K  1MQ8B                134    0.338  69.465  -0.524   0.018   0.047   0.935
E H  1MQ8B                135    0.419  76.252  -1.639   0.018   0.019   0.964
E F  1MQ8B                136    0.329  66.070  -1.355   0.058   0.017   0.925
E Q  1MQ8B                137    0.369  65.939  -0.663   0.053   0.005   0.942
E T  1MQ8B                138    0.472  65.536  -0.829   0.176   0.004   0.820
E K  1MQ8B                139    0.598 123.050  -0.523   0.406   0.004   0.590
B E  1MQ8B                140    0.214  37.333  -0.025   0.406   0.004   0.590
B S  1MQ8B                141    0.197  23.077  -0.270   0.502   0.002   0.495
E Q  1MQ8B                142    0.299  53.366   1.022   0.923   0.002   0.076
E E  1MQ8B                143    0.612 106.916   1.418   0.970   0.001   0.030
B T  1MQ8B                144    0.195  27.060   1.376   0.970   0.001   0.030
B L  1MQ8B                145    0.016   2.930   1.369   0.988   0.000   0.012
E H  1MQ8B                146    0.401  72.924   2.062   0.970   0.001   0.030
E K  1MQ8B                147    0.495 101.842   1.837   0.970   0.001   0.030
B F  1MQ8B                148    0.039   7.847   0.108   0.923   0.002   0.076
B A  1MQ8B                149    0.035   3.890  -1.369   0.782   0.003   0.216
E S  1MQ8B                150    0.654  76.602   0.341   0.502   0.002   0.495
E K  1MQ8B                151    0.416  85.468   0.751   0.246   0.004   0.750
E P  1MQ8B                152    0.522  74.015  -0.821   0.053   0.005   0.942
E A  1MQ8B                153    0.675  74.385  -1.927   0.003   0.003   0.994
E S  1MQ8B                154    0.450  52.681  -0.785   0.005   0.015   0.979
E E  1MQ8B                155    0.493  86.197   0.318   0.004   0.085   0.910
B F  1MQ8B                156    0.143  28.740   0.864   0.003   0.718   0.279
B V  1MQ8B                157    0.106  16.338  -0.144   0.001   0.959   0.040
B K  1MQ8B                158    0.087  17.937   0.634   0.001   0.959   0.040
E I  1MQ8B                159    0.290  53.576   0.707   0.001   0.900   0.099
B L  1MQ8B                160    0.041   7.489  -0.756   0.007   0.526   0.467
E D  1MQ8B                161    0.593  85.408   0.134   0.005   0.045   0.951
E T  1MQ8B                162    0.391  54.259   0.378   0.018   0.019   0.964
B F  1MQ8B                163    0.255  51.138  -0.085   0.858   0.002   0.139
E E  1MQ8B                164    0.692 120.823   0.882   0.858   0.002   0.139
E K  1MQ8B                165    0.367  75.471   1.287   0.858   0.002   0.139
B L  1MQ8B                166    0.034   6.299   0.094   0.970   0.001   0.030
E K  1MQ8B                167    0.454  93.450   2.039   0.970   0.001   0.030
E D  1MQ8B                168    0.575  82.814   2.085   0.970   0.001   0.030
B L  1MQ8B                169    0.065  11.975   0.723   0.970   0.001   0.030
B C  1MQ8B                170    0.108  15.205   0.613   0.988   0.000   0.012
E T  1MQ8B                171    0.601  83.373   2.035   0.988   0.000   0.012
E E  1MQ8B                172    0.423  73.916   2.524   0.970   0.001   0.030
B L  1MQ8B                173    0.036   6.555  -0.436   0.923   0.002   0.076
E Q  1MQ8B                174    0.399  71.279   1.346   0.923   0.002   0.076
E K  1MQ8B                175    0.708 145.553   1.339   0.782   0.003   0.216
E K  1MQ8B                176    0.518 106.491   1.863   0.694   0.003   0.303
E I  1MQ8B                177    0.377  69.763  -1.199   0.053   0.005   0.942

Explain the output.  Go back.
```

---
